# Piloting a Graduate Medical Education Point-of-Care Ultrasound Curriculum

**DOI:** 10.7759/cureus.27173

**Published:** 2022-07-23

**Authors:** Robinson M Ferre, Frances M Russell, Dina Peterson, Bita Zakeri, Audrey Herbert, Benjamin Nti, Mitchell Goldman, James G Wilcox, Paul M Wallach

**Affiliations:** 1 Department of Emergency Medicine, Indiana University School of Medicine, Indianapolis, USA; 2 Department of Radiologic Sciences, Indiana University School of Medicine, Indianapolis, USA; 3 Department of Continuing Medical Education, Indiana University School of Medicine, Indianapolis, USA; 4 Department of Internal Medicine, Indiana University School of Medicine, Indianapolis, USA; 5 Department of Family Medicine, Indiana University School of Medicine, Indianapolis, USA

**Keywords:** pilot project, residency curriculum, online medical education, graduate medical education (gme), point of care ultrasound (pocus)

## Abstract

Objective

As point-of-care ultrasound (POCUS) use grows, training in graduate medical education (GME) is increasingly needed. We piloted a multispecialty GME POCUS curriculum and assessed feasibility, knowledge, and comfort with performing POCUS exams.

Methods

Residents were selected from the following residency programs: internal medicine, family medicine, emergency medicine, and a combined internal medicine/pediatrics program. Didactics occurred through an online curriculum that consisted of five modules: physics and machine operation, cardiac, lung, soft tissue, and extended focused sonography in trauma applications. Residents completed a pre- and post-curriculum questionnaire, as well as knowledge assessments before and after each module. One-hour hands-on training sessions were held for each module. Differences between pre- and post-participation questionnaire responses were analyzed using the Wilcoxon rank sum.

Results

Of the 24 residents selected, 21 (86%) were post-graduate year two or three, and 16 (65%) were from the internal medicine program. Eighteen (67%) residents reported limited prior POCUS experience. All pre- to post-knowledge assessment scores increased (p<0.05). Statistically significant increases pre- to post-curriculum were found for frequency of POCUS use (p = 0.003), comfort in using POCUS for assessing for abdominal aortic aneurysm, soft tissue abscess detection, undifferentiated hypotension and dyspnea, cardiac arrest and heart failure (p<0.025); and competency in machine use, acquiring and interpreting images and incorporating POCUS into clinical practice (p<0.001). All participants felt the skills learned during this curriculum were essential to their future practice.

Conclusions

In this pilot, we found using a combination of online and hands-on training to be feasible, with improvement in residents’ knowledge, comfort, and use of POCUS.

## Introduction

Point-of-care-ultrasound (POCUS) is a highly useful tool used at the bedside to answer a focused clinical question and provide guidance for invasive procedures. It has been heavily integrated into the graduate medical education (GME) curricula for emergency medicine (EM) and critical care [1,2]. Other GME programs have been slower to adopt POCUS despite a large desire for training, a positive impact on improving patient care, and success rates for invasive procedures [2,3]. A 2019 study of internal medicine residency programs nationwide found that less than half of programs provide a POCUS training curriculum [4]. For pediatric training programs, POCUS training is even rarer, with only 12% of programs offering any type of POCUS curriculum [4,5]. In fact, EM is the only specialty to have a POCUS milestone as part of the core competency of residency training [6]. Simple exposure to POCUS is unlikely to be an adequate substitute for a formal curriculum, as was demonstrated in a recent study of general surgery residents who, although exposed to POCUS, were not competent in multiple critical care examinations despite planning to use them in future practice [7]. Important reasons for the lack of training broadly in GME include lack of trained faculty, equipment, societal guidelines regarding curriculum content, and confidence with image interpretation [4,5,8,9].

Multiple studies have assessed small cohorts of residents from different specialties and found it feasible to implement a POCUS curriculum during residency training, and improve POCUS knowledge, confidence, and image interpretation [5-8,10,11]. To our knowledge, no prior studies have looked at the implementation of a GME-wide POCUS curriculum. In this pilot study, we developed and implemented a multispecialty GME POCUS curriculum for graduate medical trainees in internal medicine, family medicine, emergency medicine, and combined internal medicine/pediatrics residency programs and assessed the curriculum’s feasibility as well as participants' knowledge, attitudes, and confidence with POCUS before and after the pilot.

## Materials and methods

Study design and participants

This was a prospective observational study of residents from internal medicine, family medicine, emergency medicine, and combined internal medicine/pediatric training programs at a single institution. The study was submitted to the local Institutional Review Board and a waiver of informed consent was approved.

A total of twenty-four participants from each of the four programs above were selected by their program leadership to participate in the pilot program. Of these, 16 were from internal medicine, three were from family medicine, two were from emergency medicine, and three were from the combined internal medicine/pediatric residency program. The level of training of participants was intentionally broad to provide more diverse feedback on the program. 

Curriculum

The POCUS curriculum was comprised of five online modules published on a learning management system. Modules included: core ultrasound knowledge (ultrasound physics and basic machine operation), cardiac, lung, soft tissue, and the extended-focused assessment with sonography in trauma examinations. The extended-focused assessment with sonography in the trauma exam was renamed the “dependent fluid exam” and cases were created that highlighted its use for undifferentiated hypotension outside of trauma-based scenarios. In addition to basic physics and machine operation principles, the first module also covered the scope and limitations of POCUS exams, drawing a contrast to more comprehensive and detailed studies typically ordered and performed by sonographers and interpreted by radiologists or cardiologists. 

The remaining modules focused on a specific POCUS exam. Each exam-based module was organized into five broad sections that were based on previously described POCUS sub-competencies and included: (1) module overview, (2) exam purpose and indications, (3) exam technique and images to acquire, (4) normal exam and common pathology findings, and (5) clinical case examples highlighting clinical integration [12]. Each module also includes a pre- and post-knowledge assessment required for module completion.

Participants were instructed to complete modules in a designated order over a four-month period. The first month of the pilot included two modules: core ultrasound knowledge and the dependent fluid exam. Subsequent months focused on one exam each: lung, cardiac, and soft tissue. Participants earned a Certificate of Module Completion after completing all module sections and pre- and post-knowledge assessments.

Each month, participants practiced the module exam by attending one of four scheduled, instructor-led, one-hour hands-on scan labs. Residency program leadership was consulted prior to scheduling the hands-on labs to maximize attendance and minimize conflicts with resident schedules. Participants were expected to complete one of the four scheduled hands-on labs per month. Hands-on lab attendees were required to show a print or screenshot of the Certificate of Module Completion upon entrance to the lab. The hands-on lab was excused if a conflict arose with the participant’s work duties. Participants had the opportunity to practice any missed POCUS exams at subsequent hands-on labs.

Curricular content was created by three emergency physicians with fellowship training in POCUS, a pediatric emergency physician with fellowship training in POCUS, and two multisonography credentialed Diagnostic Sonography Program faculty. All modules were peer-reviewed by other faculty with advanced POCUS training. Curricular content used a combination of text, video, still images, and illustrations. Modules were designed for an approximate one-hour completion time. 

Questionnaire and knowledge assessments

The pilot included a pre- and post-participation questionnaire designed to assess prior experience with POCUS as well as attitudes and beliefs regarding the utility of POCUS in clinical practice. A link to the pre- and post-participation questionnaire was embedded into the learning management system, and email reminders were sent to the participant group to encourage completion. A pre- and post-quiz, consisting of 10 multiple-choice questions each, were created and built into each module. 

Data analysis

Pre- and post-curriculum questionnaire responses with Likert-scale items were compared using Wilcoxon rank sum tests, as the pre- and post-curriculum questionnaires were blinded and thus could not be paired within a respondent. Pre- and post-module quiz scores were compared for differences using Wilcoxon rank sum tests. The analysis was conducted on complete data, and no method was used to replace the missing data. The mean with standard deviation and median with minimum and maximum scores are reported. A 5% significance level was used for all tests. Analyses were performed using SAS version 9.4 (SAS Institute, Inc., Cary, NC, USA).

## Results

A total of 24 residents completed the pre-curriculum questionnaire, see Table 1 for resident demographics; 88% were post-graduate year-2 (PGY2) or PGY3, and 67% were in internal medicine. Of the residents who participated in the pilot, 71% reported “very limited” or “no prior” POCUS experience, 88% completed at least one module, including pre- and post-module quizzes and the corresponding hands-on session. Participation was greatest for core ultrasound knowledge (83%), dependent fluid (83%), and lung (83%) online modules and quizzes. Participation was lowest (58%) for the soft tissue online module.

Nineteen residents (79%) participated in the hands-on fluid assessment session, 12 (50%) attended the hands-on cardiac session, 17 (71%) attended the lung hands-on session, and 14 (58%) completed the soft tissue hands-on session. Fifteen residents (62%) attended at least three hands-on sessions, with 7 (29%) completing all four. All residents attended at least one hands-on learning session, with 2 (8%) residents only attending one session (the first session-fluid assessment).

**Table 1:** Participant characteristics. PGY: post-graduate year, POCUS: point-of-care ultrasound.

Characteristics	Number (%) n = 24
Training level	
Resident PGY1	3 (12.5%)
Resident PGY2	11 (45.8%)
Resident PGY3	10 (41.7%)
Residency program	
Internal medicine	16 (66.7%)
Family medicine	3 (12.5%)
Medicine/pediatrics	3 (12.5%)
Emergency medicine	2 (8.3%)
Age	
26-30	21 (87.5%)
31-35	3 (12.5%)
Gender	
Female	7 (29.2%)
Male	16 (66.7%)
Race	
Black or African American	2 (8.3%)
Hispanic or Latino	1 (4.2)
White	20 (83.3)
Would prefer not to answer	1 (4.2)
Prior POCUS experience	
Somewhat extensive	1 (4.2%)
Somewhat limited	6 (25%)
Very limited	13 (54.2%)
No training at all	4 (16.7%)

All residents completed a pre-curriculum questionnaire, and 79% (19/24) completed a post-curriculum questionnaire. Table 2 provides a comparison of self-reported pre-curriculum POCUS utilization (within the prior six months) to post-curriculum POCUS utilization. Statistically significant increases were found from pre- to post-curriculum for the number of times POCUS was used to guide diagnosis or patient assessment (p = 0.001). Pre-curriculum, 62.5% (15/24) of participants used POCUS less than five times to guide diagnosis or patient assessment, while post-course, 89.5% (17/19) of residents used POCUS more than five times to guide diagnosis or patient assessment after the start of the course. This included two residents who used POCUS >30 times to narrow a diagnosis post-curriculum. We found no significant change in POCUS use for procedural guidance when comparing pre- to post-curriculum.

**Table 2:** Comparison of pre- and post-curriculum POCUS utilization POCUS: point-of-care ultrasound.

	Pre n = 24	Post n = 19	p-value
Number of times POCUS was used to guide diagnosis or patient assessment			<0.001*
0	5 (20.8%)		
1-5	10 (41.7%)	2 (10.5%)	
6-10	4 (16.7%)	7 (36.8%)	
11-20	4 (16.7%)	5 (26.3%)	
21-30	1 (4.2%)	3 (15.8%)	
31-40		1 (5.3%)	
>40		1 (5.3%)	
Number of times POCUS was used for procedural guidance			0.252
0	2 (8.3%)	1 (5.3%)	
1-5	6 (25%)	3 (15.8%)	
6-10	5 (20.8%)	5 (26.3%)	
11-20	7 (29.2%)	6 (31.6%)	
21-30	2 (8.3%)	4 (21.1%)	
Wilcoxon ranked sum test was used for group comparison. *Presents statistically significant results.			

When comparing pre- to post-curriculum responses, we found residents felt the following exams were more useful clinically, and they were more likely to use POCUS after completing the curriculum: undifferentiated hypotension (85% pre vs 95% post, p = 0.070), cardiac arrest (87% pre vs 95% post, p = 0.079), and heart failure (87% pre vs 90% post, p = 0.266). Residents felt they were significantly more likely to use POCUS for undifferentiated shortness of breath when comparing pre- to post-curriculum (84% pre vs 100% post, p = 0.004), with all residents finding it useful after the curriculum. We found no difference between pre- to post-curriculum for self-reported usefulness of POCUS for soft tissue abscess detection as this was high both pre and post (p = 0.914). 

Table 3 shows the resident's self-reported comfort with utilizing POCUS. Residents reported an increase in comfort using POCUS for all listed indications (p<0.001). Post-curriculum results indicated that residents felt comfortable with conducting POCUS for the following indications: undifferentiated hypotension 84%; cardiac arrest 79%; heart failure diagnosis and management 79%; undifferentiated shortness of breath 74%; soft tissue abscess detection 69%.

**Table 3:** Comparison of pre- and post-curriculum self-reported resident comfort level with POCUS based on the indication. POCUS: point-of-care-ultrasound.

		Very	Somewhat	Neutral	Somewhat not	Not at all	p-value
Undifferentiated hypotension	Pre		8 (33.3%)	1 (4.2%)	9 (37.5%)	6 (25%)	<0.001>
	Post	3 (15.8%)	13 (68.4%)	3 (15.8%)			
Cardiac arrest	Pre		7 (29.2%)	1 (4.2%)	6 (25%)	10 (41.7%)	<0.001>
	Post	5 (26.3%)	10 (52.6%)	3 (15.8%)	1 (5.3%)		
Heart failure diagnosis and management	Pre		8 (33.3%)	1 (4.2%)	9 (37.5%)	6 (25%)	<0.001>
	Post	7 (36.8%)	8 (42.1%)	4 (21.1%)			
Undifferentiated shortness of breath	Pre		2 (8.3%)	4 (16.7%)	10 (41.7%)	8 (33.3%)	<0.001>
	Post	7 (36.8%)	7 (36.8%)	5 (26.3%)			
Soft tissue abscess detection	Pre	2 (8.3%)	5 (20.8%)	3 (12.5%)	6 (25%)	8 (33.3%)	0.005*
	Post	9 (47.4%)	4 (21.1%)	2 (10.5%)	1 (5.3%)	3 (15.8%)	
Wilcoxon signed ranked sum test was used for comparison. *Indicates statistically significant results. Pre n = 24, post n = 19.							

Comparing pre- to post-curriculum residents felt more competent manipulating the ultrasound machine (41% pre vs 89% post, p<0.001), knowing anatomic structures (38% pre vs 84% post, p<0.001), obtaining POCUS images (13% pre vs 89% post, p<0.001), interpreting images (25% pre vs 79% post, p<0.001), and incorporating POCUS into clinical practice (13% pre vs 79% post, p<0.001).

All quiz scores increased from pre- to post-curriculum. Average change in scores include the following: core ultrasound knowledge 15.8% (pre 77.9% vs post 93.7%); fluid assessment 15.3% (pre 67.9% vs post 83.2%); lung ultrasound 39.5% (pre 52.1% vs post 91.6%); cardiac ultrasound 26.9% (pre 69.4% vs post 96.3%), and soft tissue ultrasound 20% (pre 60.8% vs post 80.8%), see Table 4.

**Table 4:** Comparison of pre-module and post-module quiz scores.

Pre-module and post-module quiz	n	Mean (SD)	Median (min-max)	p-value
Core ultrasound knowledge| pre-module quiz	21	7.76 (1.57)	8 (5-10)	0.002*
Core ultrasound knowledge| post-module quiz	20	9.40 (0.75)	10 (8-10)	
Fluid assessment| pre-module quiz	20	6.95 (2.03)	7.5 (4-10)	0.003*
Fluid assessment| post-module quiz	20	8.40 (1.04)	8 (6-10)	
Lung ultrasound| pre-module quiz	20	5.45 (2.25)	5 (2-10)	<0.001*
Lung ultrasound| post-module quiz	20	9.20 (0.69)	9 (8-10)	
Cardiac ultrasound| pre-module quiz	18	7.0 (2.11)	7.5 (2-10)	0.001*
Cardiac ultrasound| post-module quiz	17	9.65 (0.60)	10 (8-10)	
Soft tissue ultrasound| pre-module quiz	14	6.36 (1.49)	6 (4-10)	0.001*
Soft tissue ultrasound| post-module quiz	14	8.21 (1.05)	8 (7-10)	
Wilcoxon signed ranked sum test was used for comparison. *Indicates statistically significant results.				

In the post-curriculum evaluation, all 19 residents who responded indicated skills taught during this course were essential to their future practice (very essential: 63.2%, somewhat essential: 36.8%). Twenty-six percent (5/19) of residents indicated the knowledge and skills taught in this course exceeded their expectations, while 74% (14/19) stated it met their expectations. Likewise, 37% indicated the POCUS course as a whole exceeded their expectations, while 63% responded it met their expectations. No one indicated the knowledge and skills taught, or the course overall fell short of their expectations. In terms of rating the importance of this curriculum to their future practice, 95% of residents indicated acquiring POCUS skills was essential to their future practice, while 5% felt neutral.

## Discussion

To our knowledge, this is the first multidisciplinary POCUS pilot curriculum instituted across multiple residency programs simultaneously. Within our institution, barriers to a multidisciplinary POCUS education curriculum had previously been explored through the GME committee and included a lack of POCUS-trained faculty and faculty confidence with image interpretation. To address these concerns, an interdepartmental POCUS team developed and implemented a GME POCUS curriculum. In this study, we found that using an interdepartmental team approach decreased the limited faculty barrier to implementation of the POCUS curriculum, which other studies report [13].

We found both meaningful and statistically significant improvement in all measures of resident self-reported competence and comfort in using POCUS, as well as statistically significant improvement in their knowledge assessments. Importantly, these areas included the skills of obtaining images, interpreting images, and incorporating POCUS into existing clinical settings and scenarios. All residents who responded to the post-pilot questionnaire endorsed using POCUS to guide diagnosis or patient assessment, and how often POCUS was used also significantly increased. This finding is consistent with other programs that introduced POCUS into their GME curriculum [3,5,9,10].

Previously described specialty-specific POCUS training approaches have utilized a variety of different approaches. Some programs had a similar format to our own, using online modules followed by in-person practice sessions [3,5,9,10], while other programs solely utilized in-person instruction [2]. Several programs were centered around a one-month elective or training program [3,5,9,10], while one program had longitudinal instruction like ours, which coincided with weekly instruction across several rotations [2]. Still, other programs had the majority of their education using instruction at the bedside [10], while others relied mostly on simulated instruction and practice on normal patients [2], and a few others had a mix of simulated or online instruction and practice on patients with abnormal exams [3,5,9]. Our multidisciplinary program was mostly based on simulated instruction and practice on normal patients while providing access to ultrasound devices that could be used on patients during clinical inpatient and outpatient rotations. 

Despite these similarities, our program is unique in that it utilized a shared didactic curriculum that was developed by an interdisciplinary team of physicians with advanced training in POCUS and sonographers with experience in educational design. Furthermore, hands-on instruction utilized a pool of instructors from multiple disciplines, including: emergency medicine, internal medicine, pediatrics, pediatric emergency medicine, family medicine, sports medicine, as well as general sonography. Instruction occurred using shared training spaces and shared resources, including handheld ultrasound equipment, exam tables, linens, and other supplies. A large part of these resources, including a multidisciplinary team of instructors and equipment, was previously in place for creating and implementing a four-year longitudinal POCUS curriculum for undergraduate medical students. The implementation of both undergraduate medical education (UME) and GME POCUS training programs is part of a larger strategy by the institution to create and utilize shared resources for POCUS instruction across the entire ecosystem of medical education, from undergraduate all the way to continuing medical education for practicing physicians.

There were a few challenges in implementation. Attendance was low for soft tissue and mildly low for cardiac ultrasound examinations. This was likely due to scheduling as the curriculum was not required. To minimize scheduling conflicts, we had chief residents who were most aware of potential conflicts schedule the times for POCUS education and provided two locations to help reduce resident travel time. This barrier was also addressed by having most of the educational content available online to be viewed on demand, so they would spend the practice session solely practicing obtaining images, rather than listening to lectures. However, residents still expressed difficulty in finding time away from existing clinical and educational obligations to attend POCUS practice sessions. 

Limitations

There are several limitations to this study. Because this core curriculum was a pilot program, the subset of learners included in the program is small, from a single institution and may not be generalizable to a larger group of residents. Participants were selected by residency program leadership from those with an expressed interest in learning and helping to establish the program. It remains to be seen if this level of interest is shared among their peers or whether the pilot data is biased by selection. Furthermore, only 19 of 24 residents completed the post-pilot questionnaire, potentially skewing our results. 

Other important challenges that were foreseen when developing the pilot curriculum included limited incorporation of POCUS into the clinical environment due to a lack of equipment, particularly within the inpatient wards in the different hospitals where residents work clinically. Didactics were performed online with practice sessions utilizing peer and standardized patient scanning. While this is necessary for knowledge and skill development, future participants' views of utility and desire to participate in the program will likely be influenced by their ability to use POCUS in daily practice. 

This current study was focused on the feasibility of providing basic POCUS training to residents from several specialties. Next steps will include similar training for a larger group of residents across additional specialties to determine logistical ability to succeed. Future studies will need to objectively evaluate skill attainment, clinical use and ultimately impact on clinical care and patient outcomes.

## Conclusions

In this pilot study, we found that a combination of asynchronous online content and hands-on instruction to be feasible among a group of residents from varied specialties. The curriculum was well received and effective. There was a significant improvement in participants' knowledge and self-perceived comfort in using POCUS. Moreover, participants reported increased use of POCUS in clinical practice, particularly for diagnostic applications.
